# Knockdown of the *jph-1* gene produces altered nervous system structure and impaired muscle endurance phenotypes in *Caenorhabditis elegans*

**DOI:** 10.17912/micropub.biology.001323

**Published:** 2024-09-23

**Authors:** Rhiannon Champagne, Jose Abril, Anne Gaillard

**Affiliations:** 1 Department of Biological Sciences, Sam Houston State University, Huntsville, Texas, United States; 2 College of Medicine, Texas A&M University, Bryan, Texas, United States; 3 John Sealy School of Medicine, The University of Texas Medical Branch at Galveston, Galveston, Texas, United States

## Abstract

Calcium signaling plays an integral role in neuronal communication and muscle movement. The junctophilin family of proteins are structural components of calcium channels of the endoplasmic reticulum and are implicated in various neurodegenerative disorders. This study examined the function of *jph-1* , a gene coding for a junctophilin protein in *Caenorhabditis elegans ( C. elegans ),* by downregulating *jph-1* gene expression using RNAi through bacterial feeding. Downregulation of *jph-1* altered the physical morphology and impaired thrashing locomotion in wild-type *C. elegans .* These results are consistent with those of others in demonstrating a role for *jph-1* in muscle physiology.

**Figure 1. Knockdown of jph-1 gene expression results in altered nervous system structure and a reduced thrashing phenotype:**
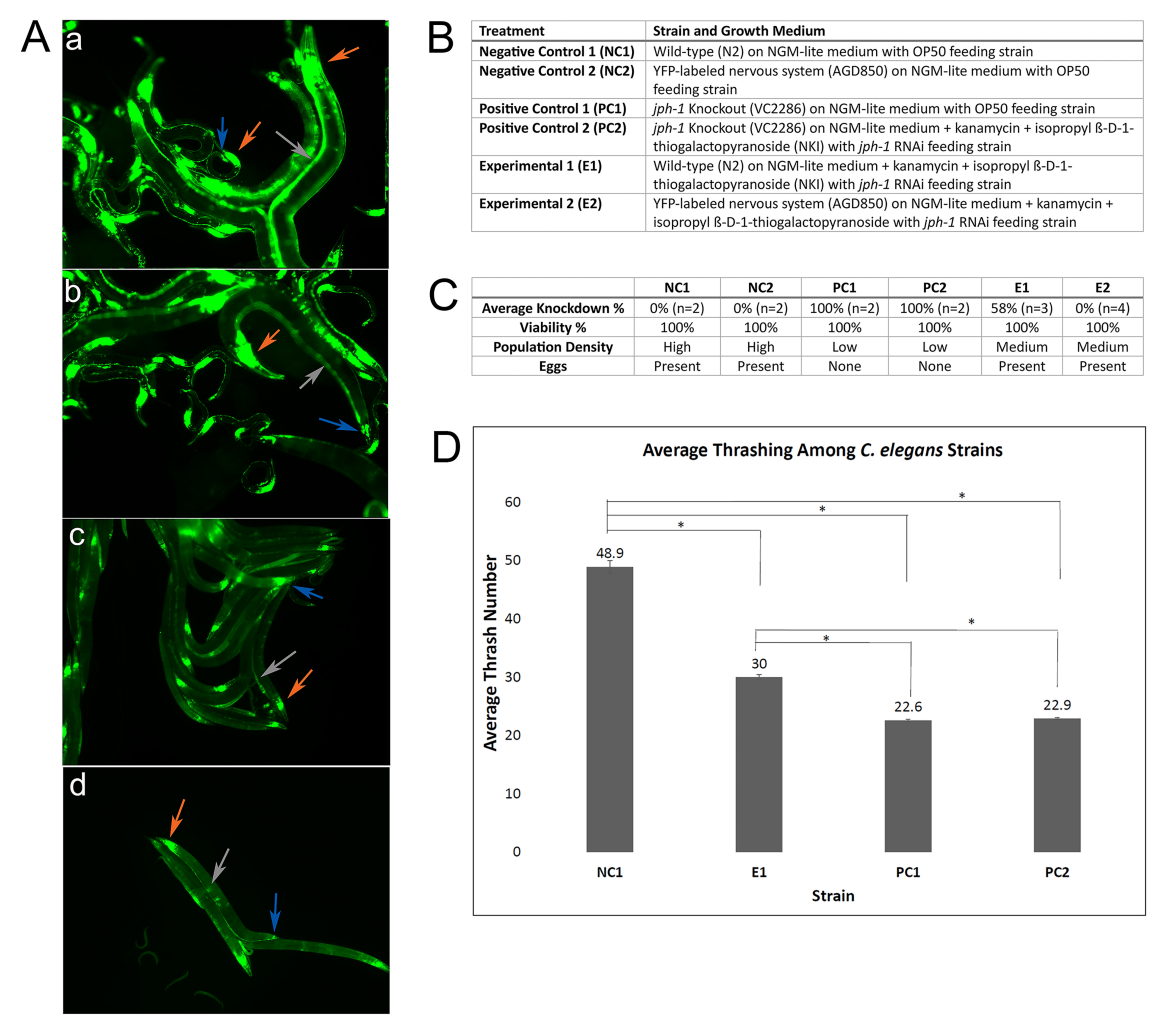
A. (a) and (b) AGD850 (YFP-labeled nervous system) strain of *C. elegans* on Nematode Growth Medium lite (NGM-lite) with OP50 *Escherichia coli* ( *E. coli* ) feeding strain exhibiting normal nervous system structure (observed day 3); (c) and (d) AGD850 strain of *C. elegans* on NGM-lite + kanamycin + isopropyl ß-D-1-thiogalactopyranoside (NKI) with *jph-1* RNAi *E. coli* feeding strain exhibiting altered nervous system structure due to RNAi knockdown. Orange arrows = head ganglion, blue arrows = tail ganglion, gray arrow = ventral cord. B. Detailed description of treatments, including *C. elegans* strains and media. C. Percent knockdown (KD%), percent viability, population density, and egg presentation for all treatments; n = number of plates observed. D. Average number of thrashes among two different strains of *C. elegans* (wild-type ( N2 ) and *jph-1* knockout ( VC2286 )) each plated on two different types of media (NGM-lite/ OP50 feeding strain; NKI/ *jph-1* RNAi feeding strain); error bars indicate the variation of average thrashes between two trials; * = P < 0.01 Tukey HSD Test; ANOVA P < 0.0001. *jph-1* knockout thrashing assay results replicate those of Piggott et al., 2021.

## Description

The junctophilin group of proteins play an important role in excitable cells, such as muscle cells and neurons (Landstrom et al., 2014; Piggott et al., 2021) . Early studies noted that junctophilin proteins were found in the membrane contact sites of the endoplasmic reticulum and the plasma membrane (ER-PM) (Garbino et al., 2009) . Junctophilin mice knockout models have elucidated the function of these proteins to be of importance for localization of calcium channels in the ER-PM areas, as well as structural support for the ER-PM contact sites (Landstrom et al., 2014) . The *C. elegans* genome contains only one junctophilin gene, *jph-1* , and its subsequent protein product is the JPH-1 protein (Piggot & Jin, 2021) . JPH-1 protein has been shown to be differentially expressed in *C. elegans* neurons and muscle cells, which denotes its role in locomotion and synaptic transmission (Yoshida et al., 2001; Piggott et al., 2021) . In humans, there are four *jph-1* orthologs that code for the junctophilin group of proteins. Deficiencies in these genes have been noted to produce Huntington-like and Charcot-Marie-Tooth-like phenotypes in mice (Landstrom et al., 2014) . They are also implicated in Huntington's disease and Charcot-Marie-Tooth disease in humans (Landstrom et al., 2014; Lehnart & Wehrens, 2022; Chivet et al., 2023) . In clinical practice, neurodegenerative disorders are key areas of interest. Understanding the role of junctophilin proteins in muscle and neuronal pathologies can pave the way for a greater understanding of the pathophysiology of these diseases, as well as potential avenues for therapeutic treatments.

RNA interference (RNAi) is a cellular mechanism that pertains to the downregulation of gene expression through interactions with mRNA transcripts and transcriptional regulation, such as DNA and histone methylation (Agrawal et al., 2003; Conte et al., 2015; Lam et al., 2015) . Studies have exhibited the induction of RNAi through the process of bacterial feeding using models such as *C. elegans* that are fed genetically-modified bacteria containing an abundance of double-stranded RNA (dsRNA) of the gene of interest (Timmons et al., 2001) . Upon ingesting the bacteria, *C. elegans* worms demonstrate the knockdown phenotype of the gene, without changes in the nucleotide sequence of their DNA. *C. elegans* is often used in RNAi research because of the relative ease of introducing dsRNA through injection, feeding, or soaking, and its unique property in which RNAi effects spread through the organism systemically (May & Plasterk, 2005) . In a reverse genetics approach, this study utilized *C. elegans* and *E. coli* containing a modified pPR244 plasmid as the bacterial RNAi feeding strain to downregulate the expression of *jph-1* in *C. elegans* . Since RNAi typically suppresses gene expression without eliminating it entirely, intermediate levels of gene expression are common, which may result in unique phenotypic effects.

To verify the construction of the *jph-1* RNAi feeding strain, plasmid DNA was extracted and sequenced. Sequencing results verified that the plasmid contained *jph-1* DNA when comparing the sequence with National Center for Biotechnology Institute databases, which yielded an E-value of 1e-47 and a percent identity of 80.98% for the *C. elegans jph-1* gene. PCR also produced a band at approximately 304 bp, which was the predicted size of the *jph-1* insert sequence.

In this study, we hypothesized knocking down *jph-1* would result in various altered phenotypes, such as locomotion variants and changes to the structure of the nervous system of *C. elegans* . The results ( Figure 1 ) demonstrated that downregulation of *jph-1* altered the physical morphology of the nervous system (E2) and impaired thrashing locomotion (E1) (p-value < 0.0001 ANOVA; p-value < 0.01 Tukey HSD), compared to wild-type (NC2 and NC1, respectively). Likewise, additional phenotypes were observed in knockdown groups (E1 and E2), such as elongation, thinner appearance, and sluggish movement (KD%, ranging from 0-58%). Viability, population density, and presence of eggs were also measured, with viability being 100% for all groups, but population density being decreased in knockdown (E1 and E2) and knockout groups (PC1 and PC2) in comparison to negative controls (NC1 and NC2). The presence of eggs was noted in both negative control (NC1 and NC2) and knockdown (E1 and E2) groups, although at a lower density in the knockdown groups. Eggs were absent in knockout groups (PC1 and PC2). Knockdown worms (E1 and E2) were also noted to move slower compared to negative controls (NC1 and NC2). Overall, phenotypic changes of the knockdown worms (E1 and E2) occurred to a lesser extent compared to the knockout worms (PC1 and PC2), as expected.

AGD850 , a YFP fluorescently-labeled nervous system worm strain, was visualized using fluorescence microscopy. RNAi-treated AGD850 worms (E2) demonstrated key differences in morphology compared to their OP50-treated counterparts (NC2). Evidence of neuronal atrophy was exhibited in E2 worms as evidenced by a dramatic decrease in fluorescence in distinct places such as the head ganglia, tail ganglia, and ventral cord ( Figure 1A ). This study was unable to confirm *jph-1* knockdown in neurons specifically; thus, *jph-1* knockdown in other cell types may be responsible for the observed changes in neuronal morphology. Indeed, RNAi is known to work poorly in neurons.

Lastly, a thrashing assay was performed to quantify the difference of locomotion between wild-type worms treated with OP50 (NC1), wild-type worms treated with *jph-1* RNAi feeding strain (E1), knockout worms treated with OP50 (PC1), and knockout worms treated with *jph-1* RNAi feeding strain (PC2). The mean thrashes per 30 seconds were 48.9 thrashes for NC1, 30 thrashes for E1, 22.6 thrashes for PC1, and 22.9 for PC2 worms. Statistical analyses of these data exhibited a significant difference among the groups using ANOVA and Tukey's Honest Significant Difference test, as shown in Figure 1D . The notion that interfering with the function of *jph-1* results in movement impairments was supported by the thrashing assay results. On average, the OP50-treated N2 worms (NC1) exhibited more thrashing movements than the *jph-1* RNAi feeding strain-treated worms (E1) and the knockout worms (PC1). Additionally, the *jph-1* RNAi-treated worms (E1) exhibited more thrashing movements than the knockout worms (PC1). This finding could be attributed due to *jph-1* 's role in calcium channel integrity in excitable cells such as myocytes and neurons, both of which are integral to normal nematode locomotion. Follow-up studies should be performed in order to build on the results of this experiment. Proteomics, in particular, could further quantify the extent of the knockdown in JPH-1 protein. In addition to examination of nervous system morphology, myocytes could also be a focal point of fluorescence microscopy. It would be interesting to examine the effect of knocking down *jph-1* in the context of muscular atrophy. Double-knockout/knockdown studies with other genes that are implicated in neurodegenerative diseases could also be useful to understand interactions amongst genes. Typically, neurodegenerative diseases implicate more than one locus, and it could be important to examine the effects of losing function of two or more genes related to neurodegeneration.

## Methods

**Amplification of the *jph-1* gene by PCR.** One tube containing an illustra™ PuReTaq Ready-To-Go™ PCR bead was obtained. A volume of 22.5 μL of *jph-1* primer mix (containing 15 pmol/µl each of Gateway cloning forward and reverse primers) was added to each tube, and the bead was allowed to dissolve for a few minutes. The primer sequences used were as follows:

5'- **GGGGACAAGTTTGTACAAAAAAGCAGGCT** GCAGCTCGTGTAACCCTCTT-3' ( **attB1** +Forward Primer)

5'- **GGGGACCACTTTGTACAAGAAAGCTGGGT** ATAACCACATAATCCCGCTCA-3' ( **attB2** +Reverse Primer)

2.5 μL of N2 genomic worm DNA was added to the tube containing the PCR bead and primer mix. The PCR tube was placed within the thermal cycler with the following programmed 30-cycle profile: a denaturing step at 94ºC for thirty seconds, an annealing step at 55ºC for thirty seconds, and a final extension step at 72ºC for sixty seconds. After cycling, the amplified DNA was stored at –20°C until gel electrophoresis was performed.

**Transformation of One Shot™ ccdB Survival™ 2T1R chemically competent cells with non-recombinant pPR244 plasmid DNA.** One vial of One Shot™ *ccd* B Survival ^TM^ 2T1 ^R^ chemically competent cells was thawed on ice prior to the transformation procedure. One µL of pPR244 plasmid DNA (10 pg) was added into the vial of One Shot™ cells and gently mixed by tapping the vial. The vial was incubated for 30 minutes on ice. The cells were then heat-shocked for 30 seconds at 42 ^o^ C without shaking. The cells were removed from the 42 ^o^ C water bath and placed back on ice for two minutes. Two hundred and fifty µL of pre-warmed S.O.C medium was added to the vial. The vial was capped tightly and shaken horizontally at 37 ^o^ C for 1 hour at 225 rpm in a shaking incubator. Once incubation was complete, 100 µL and 250 µL of the transformation cell culture in the vial were plated on two different pre-warmed selective agar plates with LB (Luria-Bertani) + 25 μg/ml chloramphenicol and 50 μg/ml kanamycin. These plates were incubated overnight at 37 ^o^ C.

**Purification of non-recombinant pPR244 plasmid DNA using QIAprep Spin Miniprep Kit.** Bacterial cell pellets were resuspended from 1-5 mL of overnight chemically competent *E. coli* liquid cultures (grown in LB + 50 μg/ml kanamycin) with 250 µL Buffer P1 containing RNase A for RNA removal. Lysis was achieved by adding 250 µL Buffer P2, with the mixture clarified and colored blue if LyseBlue was present. Neutralization then followed by addition of 350 µL Buffer N3. SDS precipitation was indicated by loss of LyseBlue coloration. The mixture was centrifuged for 10 minutes at 15,000 rpm. The supernatant was subsequently transferred to a QIAprep spin column, centrifuged for one minute, and the flow-through discarded. The column was washed with 0.5 mL Buffer PB followed by 0.75 mL Buffer PE, with centrifugation and flow-through disposal between each step as before. DNA was eluted by adding 50 µL Buffer EB to the center of the column, standing for one minute, and then centrifuging as before to collect the purified plasmid DNA.

**Formation of recombinant pPR244 plasmids via Gateway Cloning System.** Once the pPR244 plasmid was purified by DNA miniprep, a portion of the *jph-1* gene isolated via PCR as described above was inserted into the plasmid using the Gateway Cloning System and Gateway™ BP Clonase II™ . Seven µL of PCR product and 1 µL of pPR244 were incubated together with 2 µL of Gateway™ BP Clonase II™, and the reaction was incubated at room temperature for 1 hour. One µL of proteinase K was then added to the solution to stop the reaction, and the solution was vortexed briefly, followed by incubation at 37ºC for ten minutes.

**Creation of RNAi feeding strain by transformation of One Shot™ BL21(DE3) *E. coli* with recombinant pPR244 plasmid DNA containing *jph-1* DNA.** One µL of recombinant pPR244 plasmid DNA (10 pg) was added into a vial of One Shot™ chemically competent BL21(DE3) *E. coli* cells and gently mixed by tapping the vial. The vial was incubated for 30 minutes on ice. The cells were then heat-shocked for 30 seconds at 42 ^o^ C without shaking. The cells were then removed from the 42 ^o^ C water bath and placed back on ice for two minutes. Two hundred and fifty µL of pre-warmed S.O.C medium was added to the vial. The vial was capped tightly and shaken horizontally at 37 ^o^ C for 1 hour at 225 rpm in a shaking incubator. Once incubation was complete, 100 µL and 250 µL of the transformation cell culture in the vial were plated on two different pre-warmed selective agar plates with LB + 50 μg/ml kanamycin. These plates were then incubated overnight at 37 ^o^ C to select for colonies containing recombinant plasmids only.

**Verification of insert DNA via DNA sequencing and colony PCR.** The colonies produced from transformation of BL21(DE3) *E. coli* by recombinant plasmids were used to conduct another DNA miniprep as described previously. To verify insertion of *jph-1* DNA into the pPR244 plasmid, plasmid DNA was extracted from the feeding strain and submitted with a sequencing primer (M13 reverse) to Lone Star Labs (Houston, TX) for DNA sequencing. The sequencing primer was as follows: 5'-CAGGAAACAGCTATGAC-3'. Colony PCR was conducted as a second form of verification that *jph-1* was recombined within the plasmid. Five µL of the RNAi feeding strain was suspended in ultrapure water with 20 µL of *jph-1* primer mix. The cells were lysed open at 95 ^o^ C for 5 minutes, followed by PCR as before. The results of PCR were then observed using gel electrophoresis.

**Induction of RNAi with *jph-1* RNAi feeding strain and observation of phenotypes.** L4-stage worms were transferred from each stock plate to new plates containing freshly seeded bacteria. After transferring, larval stages that were transferred were verified, and worms were identified as either live, dead, or injured. Once each of the plates received their respective *C. elegans* strains, the plates were incubated upside down at 20°C. After two, three, and seven days, the plates were examined, and phenotypes were observed for each plate. Percentages of adult worms demonstrating the wild-type phenotype and the “altered” phenotypes were calculated as follows: (phenotype/total number of worms counted) * 100% = % of knockdown (KD). KD percentage was determined for the following phenotypes: long, sluggish, and thinner than wild-type. An additional phenotype assessed included qualitative observation of nervous system structure of the YFP fluorescent AGD850 strain.

**Thrashing Assay.** L4 worms were placed into a 96-well plate containing 100 µL M9 buffer solution and were observed under a dissecting microscope. The number of thrashes the worms performed in the span of 30 seconds was counted and tallied. A single thrash was described as a swimming movement with an amplitude that crossed the center of gravity of the worm. A total of 15 worms each for wild-type worms seeded with OP50 , wild-type worms seeded with *jph-1* RNAi feeding strain, *jph-1* knockout worms seeded with OP50 , and *jph-1* knockout worms seeded with *jph-1* RNAi feeding strain were observed. Two trials were performed for each worm. The average of both trials for each worm was obtained, the averages for all 15 worms were then averaged to obtain a final average for the strain, and standard deviations were calculated. Statistical analyses were conducted by using a one-way ANOVA and a Tukey HSD (Honest Statistical Difference) test. An alpha value of 0.05 was used to determine statistical significance.

## Reagents

**Table:** 

**Strain**	**Genotype**	**Available from**
N2	*Caenorhabditis elegans*	Caenorhabditis Genetics Center, University of Minnesota
AGD850	*Caenorhabditis elegans ; rmIs110* ; *uthEx557*	Caenorhabditis Genetics Center, University of Minnesota; Vilchez D, et al. Nature. 2012 Sep 13;489(7415):263-8
VC2286	*Caenorhabditis elegans ; jph-1 ( ok2823 ) *	Caenorhabditis Genetics Center, University of Minnesota; The *C. elegans* Deletion Mutant Consortium. G3 (Bethesda). 2012 Nov;2(11):1415-25.
OP50	*Escherichia coli*	Cold Spring Harbor Laboratory
One Shot™ *ccd* B Survival ^TM^ 2T1 ^R^	*Escherichia coli*	ThermoFisher
One Shot™ BL21(DE3)	*Escherichia coli*	ThermoFisher
		
**Plasmid**	**Genotype**	**Description**
pPR244 (pDONRdT7)	attL1:: *jph-1* ::attL2	246 bp sequence of *C. elegans* *jph-1* inserted between attL1 and attL2 following recombination between attB1:attP1 and attB2:attP2
